# Automated detection of Parkinson’s disease using improved linknet-ghostnet model based on handwriting images

**DOI:** 10.1038/s41598-025-12636-w

**Published:** 2025-08-21

**Authors:** P Pradeep, J Kamalakannan

**Affiliations:** Research Scholar, School of Computer Science and Engineering and Information System, Vellore Institute of Technology, Vellore, 632002 Tamil Nadu India

**Keywords:** Parkinson’s disease, Modified wiener filter, Modified PHOG, Improved LinkNet, Ghostnet, Parkinson's disease, Computer science

## Abstract

Parkinson’s disease (PD), is a neural disorder that damages movement control, which is reflected by different non-motor and motor symptoms. PD is caused by the weakening of neurons that produce dopamine in the brain, and it includes symptoms like bradykinesia (delay in movements), stiffness, and tremors. People frequently suffer from loss of motor skills when the illness worsens, which has a big influence on everyday tasks like writing. Micrographia is a disorder marked by very tiny, cramped handwriting and is one of the symptoms of PD. As a reflection of the disease’s wider motor impairments, patients may observe that their handwriting gets harder to read and control. Detecting Parkinson’s disease via handwriting images is one of the major research areas in the medical field. This research proposes an automated PD detection approach with handwriting images using an improved hybrid classification model. Primarily, a modified Wiener filter is employed for pre-processing the handwriting image. Then, modified PHOG, Deep features and Shape features are extracted. Finally, detection is performed using hybrid Improved LinkNet and Ghostnet models, termed (ILN-GNet), whose outcomes indicate if the individual is healthy or affected. From the analysis, a higher precision of 0.99 is achieved by the ILN-GNet, while existing methods attained low precision. Thus, these innovations significantly enhance early diagnosis and monitoring, enabling timely interventions before the disease progresses. Moreover, the proposed approach can contribute to remote healthcare solutions, by providing a scalable, and efficient tool for PD diagnosis.

## Introduction

Parkinson’s disease is a progressive neurological disorder that affects movement, gradually worsening over time. Key symptoms include resting tremors (involuntary shaking, often in the hands), bradykinesia (slowness of movement), and postural instability (poor balance and coordination), all of which impair fine motor skills and daily activities^1^. The main symptom of PD, a chronic and complicated neurological disorder, is movement. Although the precise origin of PD is unknown, it most likely results from a confluence of environmental variables such as pesticides, solvents, and air pollution with hereditary factors^2,3^. PD is caused by the slow degeneration of certain brain neurons that generate the neurotransmitter dopamine. Dopamine is essential for controlling coordination and movement^4–6^. PD patients have a variety of symptoms as their dopamine levels drop. The major signs of this condition are tremor, sluggish movement, rigidity of limb and volatility of body position^7,8^. The presence of such a sign could definitely influence the standard of handwriting, which is a complicated activity, which includes motor, sensory and cognitive skills. Since Parkinson’s disease severely affects fine motor control, it often causes noticeable tremors that appear as shakiness in patients’ drawings or handwriting^9^. As a result, analyzing these motor irregularities in handwriting or sketches serves as a valuable indicator for detecting PD and constitutes the base for diagnosis of the condition at its earliest stages when the severity of typical signs is minor^10–12^. Thus, changes in handwriting can be a symptom of PD.

Numerous neurodegenerative diseases that affect brain functions, like PD, have a notable impact on writing abilities^12–14^. People with PD have demonstrated evidence of micrography or dysgraphia that could be utilized as indicators to determine the possibility or severity of the disease^15–17^. Handwriting analysis is, therefore widely acknowledged as an efficient and reasonably priced approach to real-time Parkinson’s disease identification. Data about handwriting can be collected offline or online. The offline approach uses a scanner to record handwritten material on paper^2,18,19^.

Using online handwriting to identify PD presents several obstacles and hurdles. The diversity of handwriting impairments is one of the main obstacles. In actuality, each person is affected by PD in various ways and the handwriting deficits that PD sufferers experience can differ greatly^20–22^. Micrographia, or excessively tiny handwriting, is a condition that certain individuals may have. Other patients may have changes in their handwriting’s speed, pressure, or fluency. It is challenging to develop reliable handwriting characteristics for PD identification because of this diversity^2^.

A variety of diagnosis methods are required for the detection and treatment of PD, and not every healthcare facility is prepared to perform all of them. Spiral and wave diagrams are effective tools for evaluating motor skills and tremor patterns in Parkinson’s patients, offering both qualitative and quantitative insights into fine motor control^1^. The accessibility of diagnostic equipment and treatment processes might vary based on the level of resources and specialization of the healthcare facility. Certain medical facilities or neurology clinics may be the only ones with the specific knowledge and tools needed for some treatments, particularly aggressive ones like DBS surgery^23,24^. There is an urgent need for an efficient method that offers dependable, accurate PD detection. Such an approach would contribute to increasing the wellness and standard of patient life^2^.

The usage of ML methods for health data has led to DSS creation that aids medical experts in accurate decision-making. Common ML methods like KNN, RF, NB and SVM are deployed for PD detection. In particular, the development of the aforementioned techniques led to create CNN-assisted CAD models for several medical applications. CNN can learn features from the provided images, which is in contrast to hand-crafted techniques^25^. However, they do not work well with larger datasets and struggle to handle varied data types^26^. With the introduction of DL techniques like CNN and DNN, the detection of PD severity became more precise. It was discovered that CNN is more accurate than existing methods^27,28^. While pooling layers in CNNs are effective for typical image recognition tasks, they can lead to the loss of fine-grained features essential for classifying spiral drawings in Parkinson’s disease. This presents a limitation in applying standard CNN architectures in medical handwriting analysis^29^. These drawbacks encourage the development of a novel PD detection strategy in this study. Utilizing the complementing advantages of modified WF-based preprocessing and modified PHOG-based feature extraction, appropriate patterns are substantially preserved for accurate identification. Additionally, combining the improved LinkNet and GhostNet models contributes to the robust detection of PD in contrast to the existing methods.

The presented PD detection approach involves the contributions beneath.

- Proposing a modified WF for preprocessing the handwriting image using the new pixel value for the original image with the noisy image and the original image with the filtered image using the proposed Gaussian filtering, which significantly reduces the noise while preserving the edges. This results in enhancing the quality of the image for effective feature extraction.
- Employing the modified PHOG-based feature descriptor using the improved entropy for gradient computation. This improved feature effectively captures features at different resolution levels minimizes the influences of higher frequency noise and allows the model to better detect fine-grained distortions.
- Proposing a new hybrid DL model that integrates the improved LinkNet and GhostNet models for detecting PD. Leverages the capturing of multi-scale context information of the improved LinkNet and the efficient feature generation of GhostNet, leading to a well-balanced model in terms of accuracy and efficiency.
- Employing an improved LinkNet model for detection, in which the enhancements made in its architecture specifically additional layers such as WAP-BN and the MDSCM layer. This improved architecture enhances the model’s ability to learn complex spatial and channel-wise relationships and contributes to more accurate, and robust PD detection.

The reviews on PD detection are specified in Section “Literature review”. The proposed PD detection is in Section “Proposed model for Parkinson’s disease detection”. Modified WF and modified features are given under Section “Pre-processing via modified wiener filtering” and Section “Feature extraction” Improved LinkNet and Ghostnet are explained in Section “Hybrid classifiers for PD detection: Improved linknet and ghostnet”. Section “Results and discussions” and Section “Conclusions” elucidated the results and conclusion.

## Literature review

### Related works

In 2023, Naz et al*.*^30^ utilized 3 renowned PD data sets to study the issue of early PD detection using handwriting and drawing activities. The task was believed to be very challenging as there were few handwriting examples available, and the symptoms of PD might vary greatly. Various data augmentation approaches were used to increase the dataset size to accomplish better PD detection. Following that, many DCNN architectures were implemented and trained; each one’s distinct structure and layout allowed it to extract distinct prominent characteristics and aspects of the input data. Following the experimental evaluation of each CNN’s performance, the most promising feature vectors were chosen, and several early fusion techniques were used before the final classification. An ensemble of feature vectors from multiple models demonstrated significantly better generalization than a single model’s freeze vector. However, a limitation of the study is the exclusive use of the SVM classifier; despite its strong performance, other machine learning classifiers should also be explored and evaluated.

An effective DL model that helped with early PD identification was proposed by Abdullah et al*.*^31^ in 2023. The suggested model made a substantial contribution by choosing the best characteristics, which resulted in excellent performance accuracy. The KNN approach was used in GA to optimize features. The suggested innovative model leads to reduced loss and increased detection accuracy. The classification using optimized features is performed with KNN, which is computationally efficient. However, the model’s heavy reliance on feature optimization could limit its scalability and potentially affect its robustness.

Kamble et al*.*^32^ suggested a thorough scrutiny of the spirals formed by PD patients in 2021. For this, mathematical models were used to extract kinematic characteristics created for 25 individuals and 15 healthier ones. Using feature design and four ML classifiers, LR, C-SVC, KNN classifier, and ensemble model RF, the results showed a classification accuracy of almost 91% in distinguishing PD patients from healthy ones. Moreover, the model effectively identifies key kinematic features for early PD detection without complex processing. However, its reliance on a limited dataset and computational framework restricts its ability to support more comprehensive PD diagnosis.

In 2023, Konstantin et al*.*^33^ has introduced an FC approach that consisted of 3 stages: constructing the structure, picking the useful features, and parameter optimization. It was advised to use 32 variations of the approach using various metaheuristic algorithms. To diagnose PD, experiments were carried out. The handwriting of 40 persons, including 25 PS sufferers, was included in Parkinson’s HW. The handwriting exercises involved creating meanders and spirals. The handwriting of 75 persons, including 37 PD patients, was included in PaHaW. The advantages of certain realization variations in terms of prediction accuracy and interpretability were demonstrated by statistical comparisons of efficiency with other accessible classifiers, DT, and FGS. Additionally, the incorporation of a fuzzy handwriting classifier avoids the necessity of computing resources and has faster inference processing. However, there is a need for additional ML algorithms for model deployment and monitoring.

Zhu et al*.*^29^ has investigated several spiraling hand drawing characteristics of PD in 2022 and created an alternative diagnosis system based on hand drawings. First, the visual information of hand drawings accurately depicted the drawing features of individuals with PD. Second, an "Archimedes spiral hand drawing dataset" was created that was independent of the application scenario and could capture the image’s spacing, shape, and tremor features. The CC-Net was used for lowering the pooling layer. Moreover, CC-Net outperforms traditional networks in feature extraction and classification accuracy, while ensuring stable performance. However, more spiral data and multi-classification experiments across different tremor diseases are needed for further improvement.

In 2024, Hossein et al*.*^26^ has created a new technique for identifying PD with UPDRS by combining fuzzy clustering and LS-SVR. PCA and feature selection were employed to address the multicollinearity problems in the data. This study demonstrated how the suggested approach enhanced prediction through thorough assessments with existing techniques. Moreover, the PCA + FCM + LS-SVR achieve maximum precision across the test sets for Total-UPDRS and Motor-UPDRS, according to comparison results with other prediction techniques. However, using machine learning optimization techniques and adaptive heuristic search algorithms is essential to further improving the approach.

A general approach for diagnosing PD utilizing handwritten images and/or voice cues was developed by Yousif et al*.*^34^ in 2023. To diagnose PD, eight pre-trained CNN were used. 16 feature-extracting techniques were used to numerically extract features for the speech signals, which were then input into four distinct ML algorithms that were adjusted via the GSA. The segmentation of varying durations of speech signals was the basis for the novel feature extraction method used for the voice dataset. Finally, the outcomes of the experiments were gathered and documented. The incorporation of a novel feature extraction algorithm significantly enhanced detection performance. However, its main limitation is the lack of comprehensive datasets, as no publicly available PD dataset includes both handwriting and voice data from the same patients.

To enhance PD detection and classification, Mansour et al*.*^35^ presented a novel method in 2024 called QMFOFS-HCNN. The purpose of QMFOFS-HCNN was to solve the dimensionality problem and find the best feature subsets. It combined a QMO strategy for choosing features with CNN with an A-LSTM for PD recognition and classification. The Nadam optimizer was also used to optimize the selection of hyperparameters. Experimental validation utilizing benchmark datasets gave impressive findings. These numerical results highlighted how DL greatly improved the accuracy of early PD identification. It contributes to medical diagnostics by providing an effective PD detection and classification tool. The performance assessment was carried out on benchmark datasets. Nevertheless, more validation using a wider range of datasets may be necessary to ensure the technique’s practical applicability.

In 2024, Xuechao Wang et al*.*^36^ has proposed a hybrid deep learning approach that effectively combined the strengths of both LSTM and CNN for the diagnosis of Parkinson’s disease. Specifically, the LSTM component was utilized to capture time-varying features, while a CNN-based module, implemented using one-dimensional convolution, was employed to maintain low computational complexity. During data preprocessing, the forward difference algorithm was applied to extract Parkinson’s disease-related features such as resting tremor from the geometric characteristics of handwriting signals, thereby improving diagnostic accuracy with minimal processing time. Finally, the model incorporated an inference strategy that included a majority voting mechanism, resulting in highly efficient CPU inference performance. Nonetheless, a major limitation of the study lay in the small dataset size, which could potentially limit the generalizability of the findings.

In 2024, Abderrazak Benchabane et al*.*^1^ introduced an innovative method for Parkinson’s disease detection utilizing deep convolutional neural networks built upon the AlexNet architecture. Their approach centered on analyzing hand-drawn images from affected or potentially affected individuals, extracting features from these drawings for classification purposes. By merging features from two distinct types of hand drawings, the detection accuracy was notably enhanced. Nevertheless, further improvements could be achieved by exploring alternative CNN architectures, incorporating additional features, and refining the ensemble techniques used in the classification process.

### Problem statement

PD is a progressive neurological disorder with early symptoms often reflected in handwriting. Early detection is vital, but current methods face several limitations such as limited dataset diversity, model robustness, and generalizability. For instance, Naz et al*.* (2023)^30^ used ensemble DCNNs with data augmentation but highlighted challenges due to few handwriting examples and reflected the necessity of ML classifiers. Moreover, Kamble et al*.* (2021)^32^ extracted kinematic features from spiral drawings, yet their study was limited by sample size. Abdullah et al*.* (2023)^31^ achieved high accuracy using genetic algorithms and KNN but noted potential scalability issues. Additionally, Konstantin et al*.* (2023)^33^ applied fuzzy clustering and metaheuristic optimization but emphasized the need for diverse ML integration. Zhu et al*.* (2022)^29^ and Benchabane et al*.* (2024)^1^ used CNN-based methods, like CC-Net and AlexNet, showing high accuracy but requiring more varied data and better feature extraction methods. Yousif et al*.* (2023)^34^ explored both handwriting and voice data but faced limitations due to the lack of datasets with both modalities from the same subjects. Mansour et al*.* (2024)^35^ introduced QMFOFS-HCNN for feature selection and classification, yet the approach needs further validation on diverse datasets. Xuechao Wang et al*.* (2024)^36^ proposed a hybrid CNN-LSTM model, but its effectiveness is limited by a small dataset. To overcome these limitations, this research proposes an automated PD detection method using handwriting images. The majority of the current methods face challenges with limited datasets. To address these issues, the proposed model is validated using two datasets, namely the HandPD Dataset and Meander HandPD images in the HandPD dataset. By employing augmentation techniques like rotation, translation and shearing, the dimension of datasets gets increased sufficiently, which addresses the issues of limited sample size. Specifically, the incorporation of a modified Wiener filter for pre-processing and extracts modified PHOG, deep, and shape features. A hybrid model combining Improved LinkNet and GhostNet (ILN-GNet) for final classification, which significantly enhanced the detection performance. Therefore, this proposed framework aims to deliver high detection accuracy, improved generalization, and computational efficiency, which enables the proposed system to be well-positioned for deployment in practical clinical settings, potentially supporting early diagnosis.

## Proposed model for Parkinson’s disease detection

**Fig.1:**
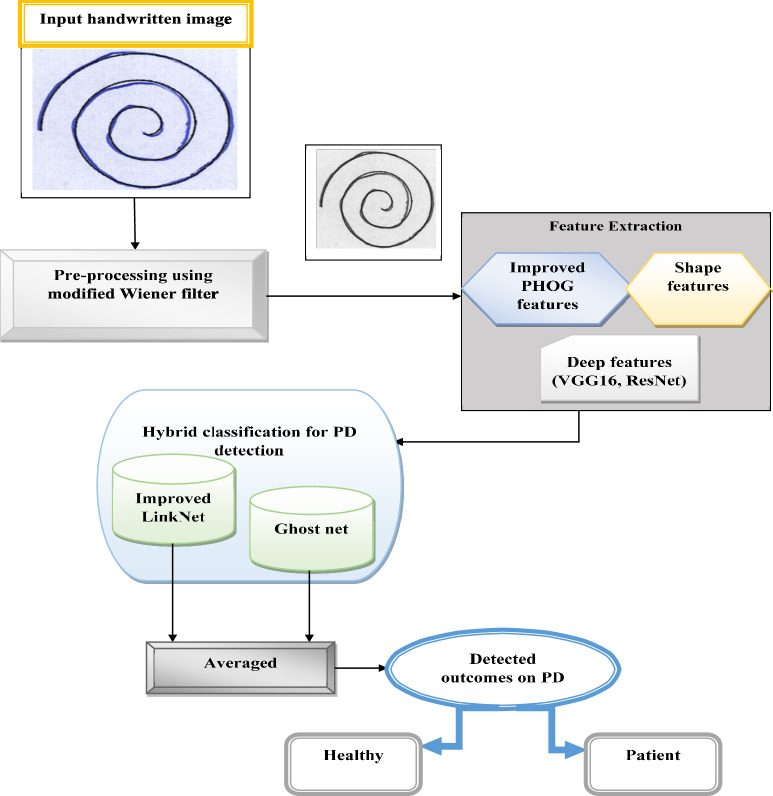
Overall Architecture of the PD detection model.

A clinical assessment of PD is made difficult due to the absence of very accurate biomarkers. The most widely used scale for evaluating both non-motor and motor symptoms of PD is the UPDRS. This scale allows doctors to evaluate the severity of motor signs in PD patients without the requirement for specialized equipment. Another issue is that the doctor’s subjective awareness has a significant impact on how this scale is evaluated. Numerous motion capture tools, including electromyography, laser displacement, accelerometers, and sensors, are available to better measure quantified tremors. Nevertheless, with the resumption of the pandemic, a simple PD screening system suitable for iPads or mobile phones would assist early identification and earlier evaluation of suspicious persons and benefit enhanced diagnosis for the patient. Many researchers have conducted significant studies on computer-aided technology in an effort to achieve an easier and more precise diagnosis of PD. However, the accuracy of PD detection remains a question. To enhance the accuracy and efficiency of the PD detection, a novel DL-based model is proposed in this research. This approach encompasses three key stages such as preprocessing, feature extraction and PD detection phases. The overall architecture of the proposed PD detection approach is shown in Fig. 1. Initially, the research begins with the preprocessing, where, the modified WF is proposed and applied to the input image to reduce noise within the image and preserve the important details for detection, leading to enhanced image quality. Subsequently, several pertinent features, including modified PHOG, Deep features and Shape features are obtained from the preprocessed image, which would offer valuable information for precise detection. Lastly, a hybrid detection model that combines the Ghostnet and Improved LinkNet models is suggested and receives the derived features as input. A more reliable and robust detection is provided by the beneficial characteristics of both models. The final detection outcomes are determined by averaging the intermediate scores from both classifiers.

### Pre-processing via modified wiener filtering

Image pre-processing involves varied methods for improving the quality of digital images, and it helps to extract pertinent information before images are further examined and processed by ML algorithms. Here, we take into consideration the input handwriting image $HI$ that has undergone pre-processing using the modified WF method.

#### Conventional WF method:

1. Calculate the PSD of noise and the original image, $HI$.
2. A mask is applied to the noise image pixel.
3. Determine local variance $\left( {\sigma^{2} } \right)$ and mean $\left( \rho \right)$.
4. Compute the new pixel value using noise power and variance, and mean.
5. The steps 2 to 4 are repeated for all noise image pixels.

Although WF reduces random noise in images, it poses certain limits. If noise PSD is erroneously computed, the existing WF might over-smooth the image or fail to eliminate the noise adequately.

While lessening the noise, it might smooth out significant details, which results in a loss of image information. Therefore, to overcome these issues, a modified WF is introduced in this work. Specifically, the incorporation of the new pixel computation for the original image with noise image and the original image with the filtered image using the proposed Gaussian filter helps to preserve the multiplicative relationships in pixel intensity values, which often better represent natural textures and subtle handwriting variations. Also, this combination not only reduces noise within the image but also significantly enhances the image quality for subsequent feature extraction processes.

#### Modified WF method:

The modified WF comprises two steps for computing the new pixel value.

- Original image with noise image.
- Original image with filtered image.

##### Original image with noise image:

1. Calculate the PSD of noise and the original image, $HI$.

2. A mask is applied to the noise image pixel.

3. Arrange the intensity of all pixels that fall under the mask.

4. Compute the median and allot it to the middle pixel of the mask.

5. Compute local variance $\left( {\sigma^{2} } \right)$ and mean $\left( \rho \right)$. The mean is computed based on the geometric mean as revealed in Eq. (1), here, $p$ and $q$ signifies the row and column of image $HI$, $k$ and $j$ signifies the count of the row and column. The variance is computed as revealed in Eq. (2).

1 $$\mu = \left( {\prod\limits_{p = 1,q = 1}^{k,j} {HI\left( {p,q} \right)} } \right)^{{{\raise0.7ex\hbox{$1$} \!\mathord{\left/ {\vphantom {1 {pq}}}\right.\kern-0pt} \!\lower0.7ex\hbox{${pq}$}}}}$$

2 $$\sigma^{2} = \left( {\frac{1}{pq}HI\left( {p,q} \right) - \rho } \right)^{2}$$

##### Original image with filtered image:

1. Enhance the brightness of the original handwriting image $HI$.

2. An improved Gaussian filter is applied. The conventional Gaussian filter is formulated as in Eq. (3), where, $c$ and $r$ refers to pixels.

3 $$GF_{c,r} = \frac{1}{{2\pi \sigma^{2} }}\exp \left( { - \frac{{c^{2} + r^{2} }}{{2\sigma^{2} }}} \right)$$

The proposed Gaussian filter is formulated as in Eq. (4).

4 $$GF_{c,r}^{{\text{Im}}} = \left[ {\frac{{\frac{1}{{2\pi \sigma^{2} }}\exp \left( { - \frac{{c^{2} + r^{2} }}{{2\sigma^{2} }}} \right) + \left[ {\frac{{c^{2} + r^{2} - 2\sigma^{2} }}{{2\pi \sigma^{6} }}.e^{{ - \left( {{\raise0.7ex\hbox{${c^{2} + r^{2} }$} \!\mathord{\left/ {\vphantom {{c^{2} + r^{2} } {2\sigma^{2} }}}\right.\kern-0pt} \!\lower0.7ex\hbox{${2\sigma^{2} }$}}} \right)}} } \right]}}{{\sqrt {c^{2} + r^{2} - 2\sigma^{2} e^{{ - \left( {c^{2} + r^{2} } \right)}} } }}} \right]$$

3. Evaluate the median $\left( {Me} \right)$ and variance $\left( {\sigma^{2} } \right)$ and mean $\left( \rho \right)$ for the filtered image.

4. Evaluate the novel value $Q\left( {p,q} \right)$ of pixel for the original image with the filtered image and noisy image as shown in Eq. (5). Here, $u$ signifies noise variance.

5 $$\begin{gathered} Q\left( {p,q} \right) = \left[ {med + \frac{{\sigma^{2} - u^{2} }}{{\sigma^{2} }}\left( {HI\left( {p,q} \right) - med} \right)} \right] + \hfill \\ \left[ {med_{1} + \frac{{\left( {\sigma^{2} } \right)^{\prime} - u^{2} }}{{\left( {\sigma^{2} } \right)^{\prime}}}\left( {HI\left( {p,q} \right) - \rho } \right)} \right] \hfill \\ \end{gathered}$$

5. The steps of the original with noise and the original with filtered image are repeated for all noise image pixels.

The pre-processed image is indicated by $HI^{p}$.

Thus, the modified WF is capable of removing the noise, whereas the edges are preserved. In addition, the modified WF reduces the distortions. The modified WF could conserve edges and significant details and effectively lessen the noise.

### Feature extraction

In image processing, feature extraction is crucial. Digital images can have factors like motion, shapes, or edges that are detected during feature extraction. Following their identification, the data may be processed to carry out several image analysis tasks. The distinctive features listed below are obtained from this study:

- Modified PHOG features
- Shape features
- Deep features (VGG 16 and ResNet)

The combination of modified PHOG features, shape features, and deep features from VGG16 and ResNet creates a comprehensive, multi-layered feature set that significantly improves the accuracy of PD detection. The modified PHOG features capture information across different spatial resolutions, enhancing gradient calculations by reducing the impact of high-frequency noise and emphasizing significant gradient variations. Meanwhile, the shape features provide crucial global geometric insights, that are key for distinguishing PD-related handwriting abnormalities. The deep features extracted from VGG16 and ResNet offer robust hierarchical representations, enabling the model to detect intricate and abstract patterns. Together, these diverse and complementary features enhance the model’s ability to detect PD with greater precision and ensure more reliable detection.

#### Modified PHOG features

The PHOG model^37^ splits the image, $HI^{p}$ at various resolutions to districts depending upon spatial pyramid matching. The doubling of divisions in the axis direction is repeated by splitting $HI^{p}$ into fine spatial grids. In PHOG, the derivative mask is deployed in the vertical and horizontal directions of $HI^{p}$ to calculate the gradient. Especially, this model requires gray level image filtering for kernels like, $$K_{X} = \left[ { - 1\,\,0\,\,1} \right]\,\,and\,\,K_{Y} = \left[ \begin{gathered} \,1 \hfill \\ \,0 \hfill \\ - 1 \hfill \\ \end{gathered} \right]$$ and the derivatives of *X* and *Y* are achieved with convolution operations namely $U_{X} = HI^{p} * K_{X} \,\,and\,\,U_{Y} = HI^{p} * K_{Y}$. The orientation and magnitude of the gradient are assessed as in Eq. (6) and (7).

6 $$U_{O} = \arctan \frac{{U_{Y} }}{{U_{X} }}$$

7 $$U_{G} = \sqrt {\left( {U_{X}^{2} + U_{Y}^{2} } \right)}$$

While traditional PHOG features are good at explaining the image’s spatial information, they primarily focus on local gradient details and are unable to effectively capture global context details without additional features. Also, the conventional PHOG is based solely on gradient orientation and magnitude, which makes it highly sensitive to noise and artifacts. This leads to poor capturing of non-linear distortions, as it assumes relatively stable gradient behavior. In order to address these shortcomings, a modified PHOG feature is proposed based on the incorporation of improved entropy in the gradient operation, which allows for focusing on high-information regions. Additionally, the modified PHOG is more resilient to noise and slight distortions, ensuring clearer and more trustworthy feature maps and improving the precision of PD identification.

**Modified PHOG:** As per modified PHOG, the gradient operation is modelled depending upon improved entropy as shown in Eq. (8) and (9).

8 $$U_{X} = HI^{p} * K_{X} \,\, + IE$$

9 $$U_{Y} = HI^{p} * K_{Y} + IE$$

1. Computation of improved entropy for each gradient image:

- Evaluate the improved entropy of the horizontal gradient image $U_{X}$.
- Evaluate the improved entropy of the vertical gradient image $U_{Y}$.

The conventional Shannon entropy is given in Eq. (10).

10 $$En = - \sum\limits_{i = 1}^{k} {P\left( {HI_{i}^{p} } \right)} \log_{b} P\left( {HI_{i}^{p} } \right)$$

The improved Shannon entropy used in modified PHOG for gradient computation is given in Eq. (11). In Eq. (11), $\left| M \right|$ signifies the cardinality of the focal component $M$, $\Theta$ signifies the FOD.

11 $$En^{{\text{Im}}} = \frac{{\left[ { - \sum\limits_{M \subseteq \Theta } \begin{gathered} a\left( M \right)\log_{2} \frac{{a\left( M \right) + 2^{\left| M \right|} - 1}}{{P\left( {HI_{i}^{p} } \right)\log_{b} P\left( {HI_{i}^{p} } \right)}} + \hfill \\ \sum\limits_{M \subseteq \Theta } {a\left( M \right)\log_{2} \left( {2^{\left| M \right|} - 1} \right)} \hfill \\ \end{gathered} } \right]}}{{\left[ {\sum\limits_{M \subseteq \Theta } {a\left( M \right)\log_{2} a\left( M \right) + \frac{1}{{1 + e^{{ - \left( {\log_{2} a\left( M \right)} \right)}} }}} } \right]}}$$

2. Comparing the improved entropy values:

- After evaluating the improved entropy of $U_{X}$, the threshold should be set as the mean of entropy.
- Higher entropy in $U_{X}$ denotes a high difference in the horizontal edge, while higher entropy in $U_{Y}$ denotes a high difference in the vertical edge. If entropy is similar in both gradients, it denotes that edge info is distributed evenly in both orientations.
- Select the high entropy value that sums with $U_{X}$ and $U_{Y}$. Here, the gradient entropy quantifies how much difference persists in every gradient image.
- Now, by summing the higher entropy value to the gradient mean, the gradient containing the more directional info can be enhanced.

The modified PHOG features denoted by $PHOG^{M}$ captivates features at varied spatial resolution levels. It further improves the accuracy of gradient computation by lessening the influence of higher-frequency noise and highlighting remarkable gradient disparities. Thus, compared to existing PHOG, the modified PHOG features aid in gathering fine details needed for PD detection.

#### Shape features

Shape features $SF$^38^$!$ are extracted in this proposed work to improve the understanding of object geometry in the image, $HI^{p}$. Features like area, epsilon, hull and perimeter are specifically taken into account. Epsilon assesses the shape’s intricacies and imperfections to determine how closely a component’s shape resembles its usual form. The perimeter establishes the overall length of the border, while the area determines the item’s size. The convex hull provides the lowest convex border that may surround the shape. When combined, these form elements are essential for image evaluation and retrieval because they enable accurate object comparison and characterisation based on geometric aspects.

#### Deep features

Deep features $\left( {DF} \right)$ are obtained from $HI^{p}$ by applying the ResNet and VGG 16 models, are considered in the suggested study for PD identification.

***ResNet:*** The ResNet model incorporates residual connections that allow the network to go deeper without suffering from the vanishing gradient problem. This enables ResNet to capture more complex and abstract visual representations by learning deeper hierarchical features for precise detection. Convolutional, pooling, normalizing, and FCL are all included in the ResNet-50 model^39^. By obtaining residual functions that allow the network to gather a wide range of attributes from input images, including both specific low-level and high-level data, residual blocks facilitate optimization. Features are extracted using convolutional layers, filtered using residual blocks, the spatial extent is reduced using max pooling, and non-linearity is addressed using ReLU. These specific attributes are then used by the final FCL to categorize the image.

***VGG 16:*** It^40^ is a DCNN architecture noted for its simplicity and efficacy in image classification. VGG16 is known for its deep, uniform architecture, this design enables the model to learn hierarchical features at multiple levels, from basic edges and textures at lower layers to complex, high-level patterns at deeper layers. This characteristic allows the model to capture both fine details crucial for accurate detection. Its architecture comprises 16 weight layers: 13 con layers and 3 FCL grouped into five conv blocks. To enable VGG-16 to acquire hierarchical features from simple edges to intricate patterns, each block reduces spatial dimensions by using max pooling and raises the filter count from 64 to 512. To capture micro features, the network uses tiny 3 × 3 filters in conv layers. In VGG16, the input moves via a sequence of conv layers and max pooling layers. The flattened feature maps are categorized by FCLs, and the learning process is enhanced by the non-linearity added by the ReLU activation.

The final feature $Ff = \left[ {PHOG^{M} \, + SF + \,\,DF} \right]$ is an inclusive grouping of modified PHOG, deep features and shape features. This varied set of features permits robust detection by capturing thorough information from several aspects of $HI^{p}$.

### Hybrid classifiers for PD detection: Improved LinkNet and Ghostnet

The features $\left( {Ff} \right)$ are subjected to hybrid classifiers such as improved LinkNet and GhostNet for identifying the PD. Rather than the application of single classifiers, the application of hybrid classifiers will result in better outcomes. Integrating the improved LinkNet and GhostNet models in the proposed ILN-GNet framework offers a powerful synergy that enhances the accuracy, efficiency, and robustness of PD detection. The improved LinkNet excels in detailed spatial feature extraction and multi-scale context learning through its advanced encoder-decoder structure, and modules like MDSCM and WAP-BN, making it highly effective at capturing subtle handwriting variations. On the other hand, GhostNet contributes lightweight, high-efficiency feature extraction by generating more feature maps through simple linear operations, significantly reducing computational complexity without sacrificing performance. Combining the improved LinkNet with GhostNet models offers an efficient and compact representation of learning, and effectively distinguishing PD-affected handwriting. The performance of improved LinkNet and GhostNet is examined using “HandPD Dataset^41^ and Meander HandPD images in the HandPD dataset^42^”. The HandPD Dataset detects the individual as “healthy or patient”.

#### GhostNet

The features $Ff$ are subjected to the GNet architecture for the detection of PD. However, the traditional deep models may provide high accuracy but often come with large numbers of parameters and heavy computational demands. In contrast, the GhostNet model strikes a balance by maintaining competitive accuracy while drastically reducing computational costs. Hence this model has been employed in this research for detection. GNet^43^ comprises a novel Ghost module that uses low-cost operations to produce additional feature maps. This novel NN efficiently generates more feature maps with fewer variables and computations. The implementation of this module consists of two parts. Using a conventional convolutional computation, GNet first generates feature maps with fewer channels. Next, it uses a straightforward procedure to generate more feature maps. Finally, it combines several feature maps to produce a new output. The GBM in GNet is separated into two categories based on stride. Two Ghost modules make up the GBM design when stride = 1, which is described using conventional residuals. To add more channels, the initial module serves as an extension layer. In order to link the outputs and inputs of these two Ghost modules, the second module first decreases the number of channels to match the shortest path. When stride = 2, the GBM has the conventional bottleneck structure’s layout, and when stride = 1, it keeps its structural features. The ReLU nonactivation operation and BN are used by the subsequent layers following the second Ghost module. The final result from GNet is displayed as $Gn^{o}$.

#### Improved LinkNet

The features $Ff$ are employed in the LinkNet classifier for PD detection. 3 encoder and decoder components make up the LinkNet architecture^44^. They both often carry out ReLu activations, BNs, and convolution. While the BN helps to standardize training and attain a high rate of convergence, the Conv layer is utilized to detect spatial patterns. The normalized output is then sent into the Leaky ReLU for training the complex features and the average pooling layer to reduce the spatial dimensions. Similarly, encoder block 2 applies Conv, BN, average pooling, and mix pooling layers to encoder block 1 to enhance the accuracy of PD detection. The results of encoder block 2 are then sent to encoder block 3, which includes a conv layer and BN.

Even while ordinary LinkNet generates fine-detected outputs, the recurrent striding and pooling techniques utilized in LinkNet lower feature resolution. This makes it possible that some information will be lost. Therefore, to prevail over such problems, we introduce an improved LinkNet model for PD detection. Thereby, an improved LinkNet architecture is proposed with varied modifications in its structure. The proposed LinkNet architecture includes extra layers such as mixed pooling, average pooling, encoder 1 followed by conv, BN, Leaky ReLU, encoder 2 followed by conv, BN, ELU, encoder 3 followed by conv, BN, swish, decoder 1, 2 and 3 followed by WAP-BN layers. Particularly, the MDSCM layer is included in the improved LinkNet architecture. This improved architecture enables the model to learn complex, non-linear handwriting patterns more effectively. The use of mixed and average pooling improves feature preservation by balancing edge detection and texture retention, while the decoder stages incorporate WAP-BN to enhance reconstruction accuracy and training stability. Notably, the integration of the MDSCM allows efficient multi-scale feature extraction with reduced computational cost, making the model both lightweight and powerful. Overall, these structural improvements enhance the model’s ability to capture subtle variations in handwriting, leading to more accurate and robust PD detection.

**Fig.2:**
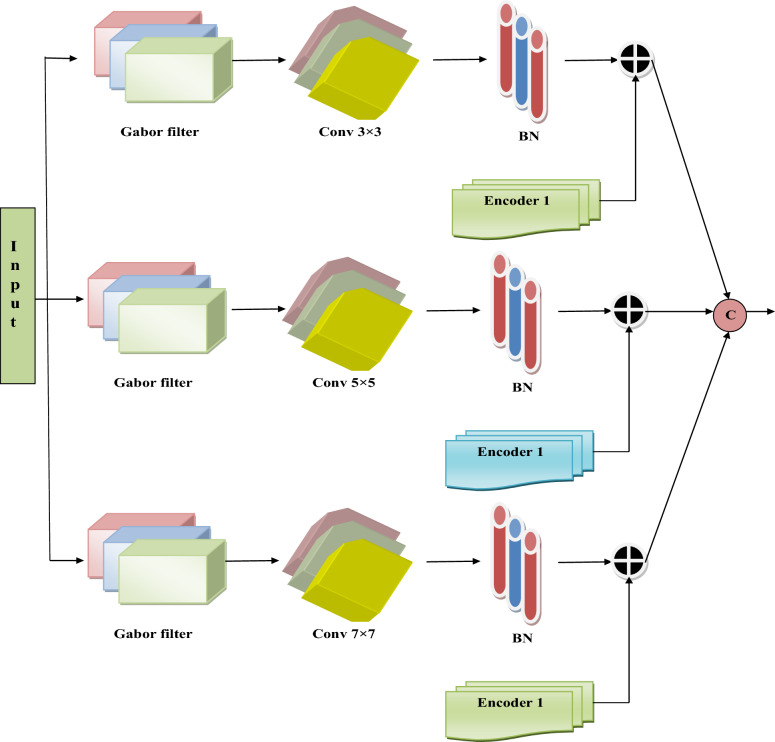
Architecture of Suggested MDSCM module.

**MDSCM layer:** The MDSCM layer includes 3 Gabor filters, 3 conv layers with strides 3 × 3, 5 × 5 and 7 × 7, 3 BN layers and 3 encoders. Each Gabor filter output is connected to the Conv layer with each stride. The output from the conv layer is passed as input to the BN layer. The output from the BN layer and each encoder block is XOR-ed, and the outputs are concatenated to get the final output. The MDSCM architecture is shown in Fig. 2.

**Proposed WAP-BN computation:** The conventional BN is modelled as in Eq. (12), where, $Z_{i}$ denotes the decoder output,$\delta_{B}$ represent the mean value of $Z_{i}$, $\varepsilon$ refers to a constant deployed for numerical constancy.

12 $$\hat{Z}_{i} = \frac{{Z_{i} - \delta_{B} }}{{\sqrt {\sigma_{B}^{2} + \varepsilon } }}$$

However, for better stabilization of parameters, instead of the existing BN, we propose weighted average pooling-based BN in the modified LinkNet design.

The proposed WAP-BN is modelled as in Eq. (13), where, $S_{J}$ signifies weighted average pooling^45^ that is formulated as given in Eq. (14).

13 $$WAP - BN = \frac{{\left( {Z_{i} * S_{J} } \right) - \left( {\delta_{B} * S_{J} } \right)}}{{\sqrt {\left[ {\sigma_{B}^{2} + \frac{1}{{\left( {1 + e^{{ - \left( {S_{J} } \right)}} } \right)}}} \right] + \varepsilon } }}$$

14 $$S_{J} = \left( {1 - \gamma } \right)\frac{1}{{r_{J} }}\sum\limits_{{i \in r_{J} }} {A_{i} }$$

In Eq. (14), $\gamma$ is set as 1, $B$ refers to a parameter, $A_{i}$ refers to the feature value at the position $i$ inside the pooling area $r_{J}$.

**Fig.3:**
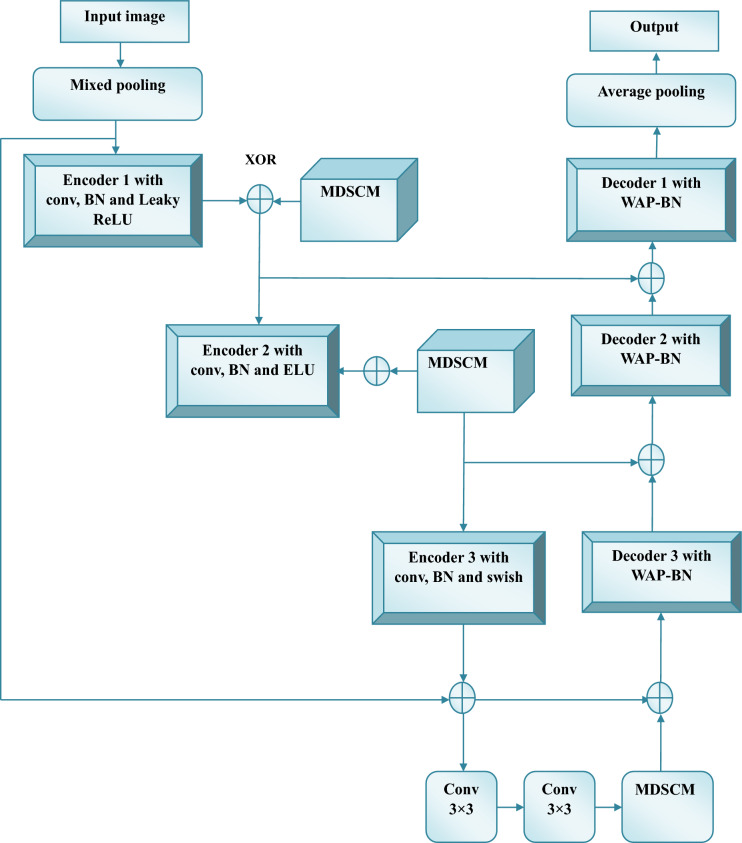
Proposed improved LinkNet Architecture.

The improved LinkNet model is highly useful for utilizing global data at different scales. When compared to the current LinkNet model, the modifications made to the upgraded LinkNet model reduce processing costs and preserve aggregate multi-scale context information. Additionally, improved LinkNet speeds up training and makes the procedure much more efficient. Figure 3 shows an architecture of the improved LinkNet.

The final improved link output is signified by $ILn^{o}$.

The outcomes from improved LinkNet $\left( {ILn^{o} } \right)$ and GhostNet $\left( {Gn^{o} } \right)$ are subjected to mean and final PD-detected outcomes are attained as “healthy or patient”.

## Results and discussion

### Simulation procedure

The ILN-GNet model developed for PD detection was implemented using Python. Additionally, to run the simulation, we employed a system that had a 11th Gen Intel(R) Core (TM) i5-1135g7 CPU operating at 2.40 GHz and 2.42 GHz and 16.0 GB (15.7 GB usable) of RAM. An × 64-based CPU running a 64-bit operating system was the system type. The valuation was done for ILN-GNet over LinkNet, GhostNet, GoogleNet, AlexNet^1^, EfficientNet, CNN^30^, Ensemble^12^, LSTM-CNN^36^ and XGBoost^46^. The study data was attained using HandPD Dataset (dataset 1)^41^ and Meander_HandPD images in HandPD dataset (dataset 2)^42^.

### Dataset description

#### HandPD dataset (dataset 1)

The HandPD dataset consists of handwritten tests from two groups of people: the (i) Healthy Group and the (ii) Patient Group, which is made up of people with Parkinson’s disease (PD). Of the 92 participants in the sample, 74 are patients (patients group) and 18 are healthy (Healthy group). A synopsis of each group can be found below:

Healthy Group: There were six men and twelve women, representing ages 19 to 79 (average age: 44.22 ± 16.53 years). Two of those people are left-handed, and sixteen are right-handed.

The patient group consisted of 15 females and 59 males, aged 38 to 78 (average age: 58.75 ± 7.51 years). Of those people, 69 are right-handed and 5 are left-handed. Consequently, the entire dataset consists of 736 images labeled in two groups: 72 images in the healthy group and 296 images in the patient group. This results in a dataset including 368 images from each drawing, such as meanders and spirals. After the application of augmentation techniques such as rotation, translation and shearing, the total number of images was increased to 1240, which were distributed into the healthy group has 648 images and the patient group has 592 images. The images are labeled as follows: ID_EXAM-ID_IMAGE.jpg, in which ID_EXAM stands for the exam’s identifier, and ID_IMAGE denotes the number of the image of that exam”.

#### Meander_HandPD images in HandPD dataset (dataset 2)

**Fig.4:**
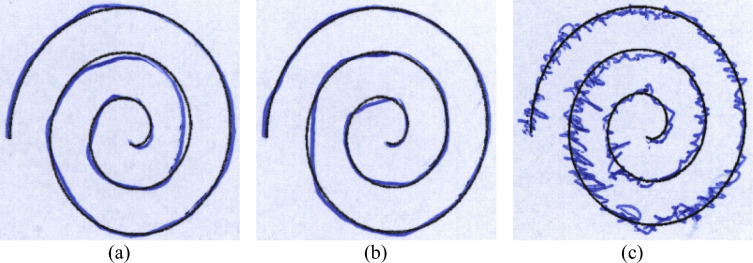
Sample for PD detection using handwriting images for dataset 1 (**a**) sample 1 (**b**) sample 2 and (**c**) sample 3.

**Fig.5:**
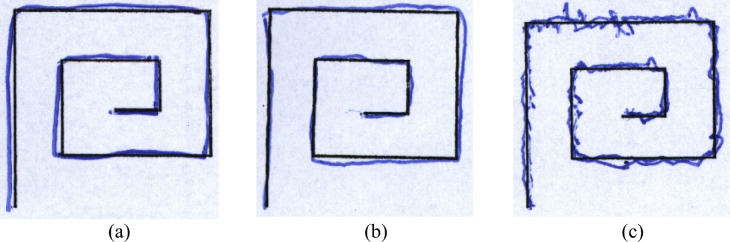
Sample for PD detection using handwriting images for dataset 2 (**a**) sample 1 (**b**) sample 2 and (**c**) sample 3.

Handwritten samples from two groups such as a Healthy Group and a Patient Group, consisting of people with PD diagnoses make up the HandPD dataset. These samples of handwriting were acquired at São Paulo State University’s Botucatu Medical School in Brazil. Participants were instructed to complete a form containing four spiral patterns and four meander patterns. These sections were later cropped from the forms and saved as individual JPEG images. The dataset contained 368 images, categorized into two classes: MeanderControl (72 images), and MeanderPatients (296 images). To expand the dataset, data augmentation techniques (rotation, translation and shearing) were applied, increasing the total number of images to 1,240. Post-augmentation, the distribution included 648 images for MeanderControl and 592 images for MeanderPatients. The samples of handwriting images for PD detection for Dataset 1 and Dataset 2 are shown in Fig. 4 and Fig. 5 respectively.

### Pre-processing analysis

**Fig.6:**
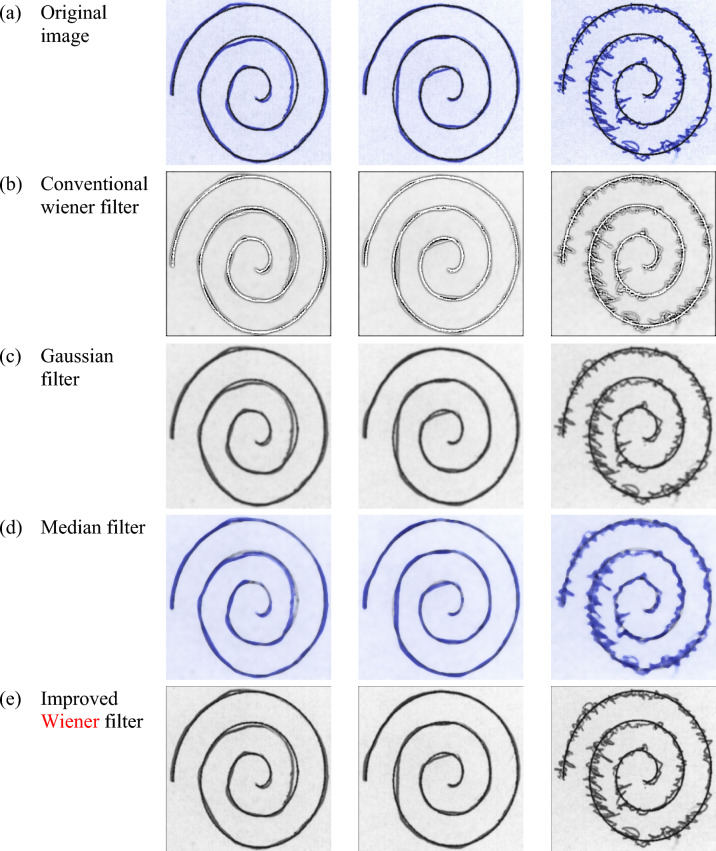
Sample for PD detection using handwriting images (**a**) Original image (**b**) Traditional wiener filter (**c**) Gaussian filter (**d**) Median filter and (**e**) Improved Wiener filter for dataset 1.

**Fig.7:**
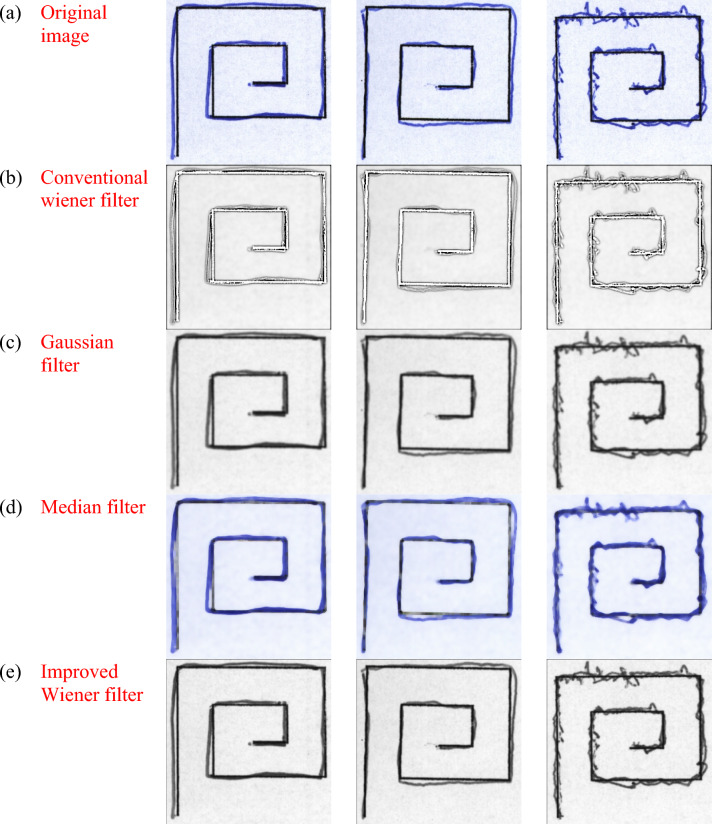
Sample for PD detection using handwriting images (**a**) Original image (**b**) Traditional wiener filter (**c**) Gaussian filter (**d**) Median filter and (**e**) Improved Wiener filter for dataset 2.

The study of the modified WF used to pre-process the handwriting images using Dataset 1 and Dataset 2. For accurate detection, the handwriting images utilized for PD detection should have a high resolution. The visual representation of the conventional WF, Gaussian filter, median filters, modified WF for datasets 1 and 2 are illustrated in Fig. 6 and Fig. 7 respectively.

Table 1**.** illustrates the preprocessing analysis in terms of PSNR and SSIM metrics regarding datasets 1 and 2. After preprocessing, PSNR is frequently used to assess an image’s quality. Greater PSNR values show that there is reduced noise and distortion from the filter, bringing the processed image closer to the original. SSIM, on the other hand, takes into account structural details like texture, contrast, and brightness when determining an image’s perceived quality. The model successfully maintains the structural details when the SSIM value is high. The model can detect the PD more precisely when higher PSNR and SSIM are achieved. The PSNR and SSIM attained using modified WF are high, around 32.97 and 0.946, respectively. On the other hand, extant WF, Gaussian and median filters attain lower PSNR and SSIM values. Using dataset 2, the Improved Wiener filter achieved a greater value on PSNR, about 33.450, meanwhile Conventional Wiener filter = 30.540, the Gaussian filter = 23.640 and the Median filter = 26.480. Additionally, the Improved Wiener filter acquired better values on SSIM, about 0.957, in contrast to the traditional methods. Therefore, the enhancements carried out in the improved WF offer high PSNR and SSIM, which translate directly into better input quality for the hybrid ILN-GNet model. As a result, the ILN-GNet model can more effectively distinguish between healthy and PD-affected individuals, leading to improved detection accuracy.

**Table 1:** Pre-processing Analysis for Datasets 1 and 2.

Dataset 1			Dataset 2		
Methods	PSNR (dB)	SSIM	Methods	PSNR (dB)	SSIM
Traditional Wiener filter	29.560	0.906	Conventional Wiener filter	30.540	0.917
Gaussian filter	25.120	0.857	Gaussian filter	23.640	0.826
Median filter	27.860	0.876	Median filter	26.480	0.857
Improved Wiener filter	32.970	0.946	Improved Wiener filter	33.450	0.957

### Performance analysis

**Fig.8:**
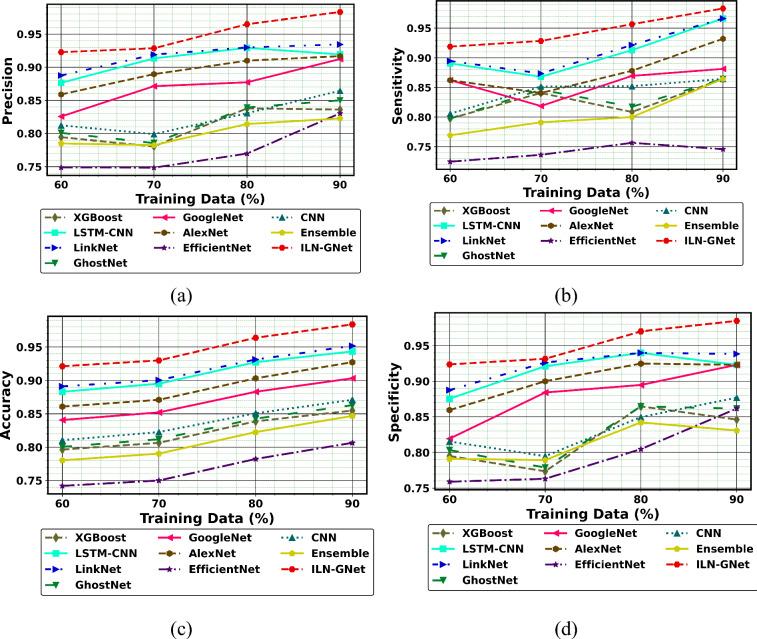
Performance of PD detection with handwriting images using ILN-GNet over traditional method on (**a**) Precision, (**b**) Sensitivity, (**c**) Accuracy, (**d**) Specificity using dataset 1.

**Fig.9:**
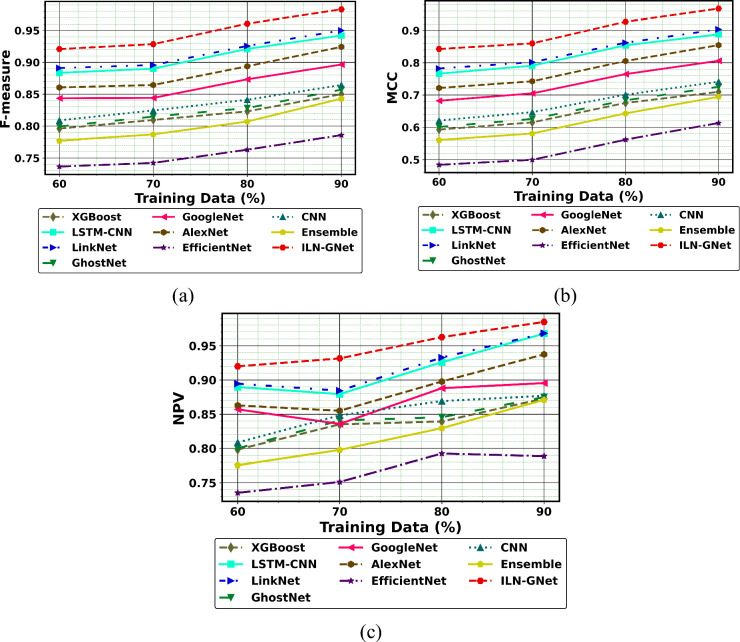
Performance of PD detection with handwriting images using ILN-GNet over traditional method on (**a**) F measure, (**b**) MCC, (**c**) NPV for dataset 1.

**Fig.10:**
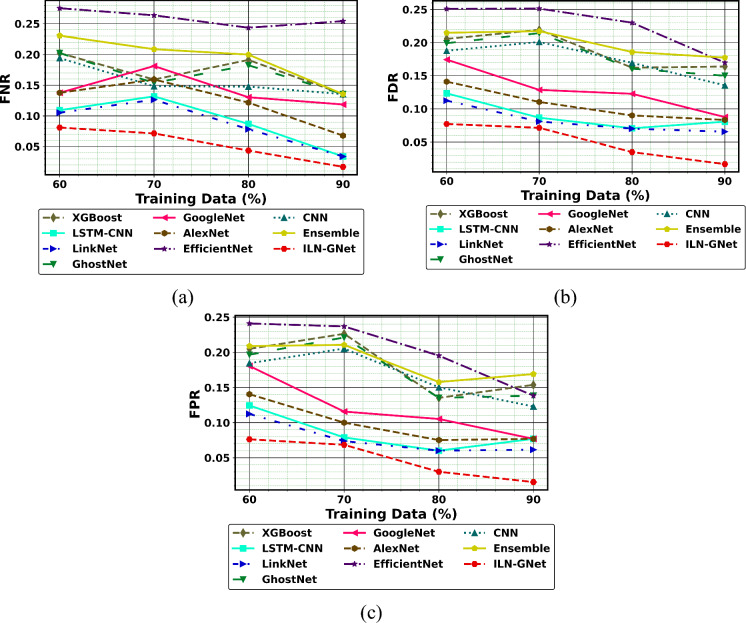
Performance of PD detection with handwriting images using ILN-GNet over traditional method on (**a**) FNR, (**b**) FDR and (**c**) FPR for dataset 1.

Figures 8, 9, and 10 illustrates the performance comparison of ILN-GNet over the conventional method using dataset 1. As per the outcomes, ILN-GNet for PD detection attains better precision than LinkNet, GhostNet, GoogleNet, AlexNet, EfficientNet, CNN^30^, Ensemble^12^, LSTM-CNN^36^ and XGBoost^46^. The ILN-GNet model achieved a higher precision of 0.99 at TD = 90%, where LinkNet, GhostNet, GoogleNet, AlexNet, EfficientNet, CNN^30^, Ensemble^12^, LSTM-CNN^36^ and XGBoost^46^ got minimal precision values. Here, hybrid classifiers such as improved LinkNet and GhostNet are used for identifying the PD. Several modifications to structures are suggested for the improved LinkNet architecture. The improved LinkNet model includes the MDSCM layer in particular. Furthermore, rather than using BN layers, the decoder uses WAP-BN layers. Comparing the improved LinkNet model to the conventional, the modifications made reduce processing costs and preserve aggregate multi-scale context information. The negative measure, FDR using ILN-GNet for PD detection is less than 0.02, while LinkNet, GhostNet, GoogleNet, AlexNet, EfficientNet, CNN^30^, Ensemble^12^, LSTM-CNN^36^ and XGBoost^46^ attain high FDR values. Likewise, the neutral and positive metrics using ILN-GNet for PD detection are high, while, negative metrics using ILN-GNet attain low values. Therefore, faster training and more process effectiveness are achieved by the combination of improved LinkNet and Ghostnet.

**Fig.11:**
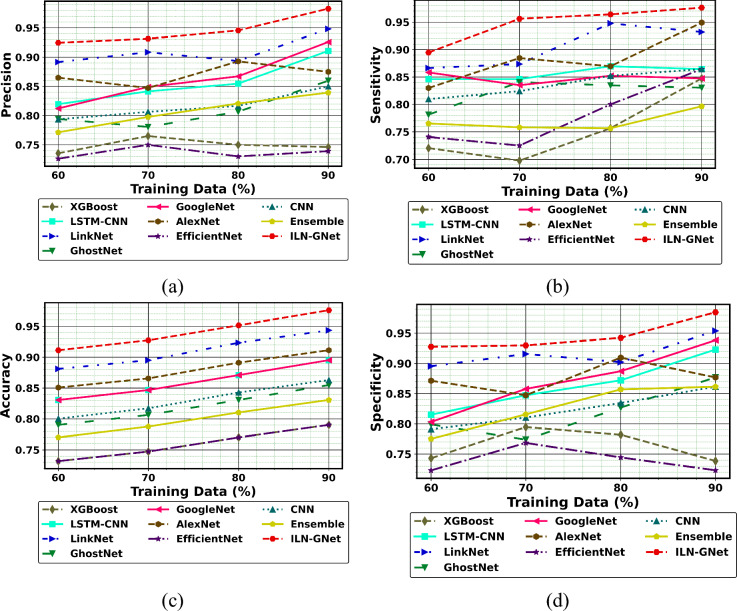
Performance of PD detection with handwriting images using ILN-GNet over traditional method on (**a**) Precision, (**b**) Sensitivity, (**c**) Accuracy, (**d**) Specificity for dataset 2.

**Fig.12:**
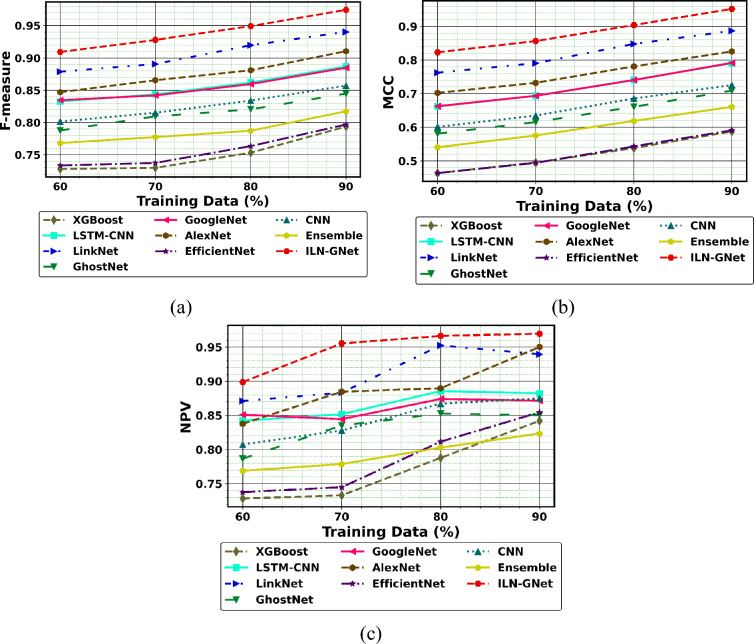
Performance of PD detection with handwriting images using ILN-GNet over traditional method on (**a**) F measure, (**b**) MCC, (**c**) NPV for dataset 2.

**Fig.13:**
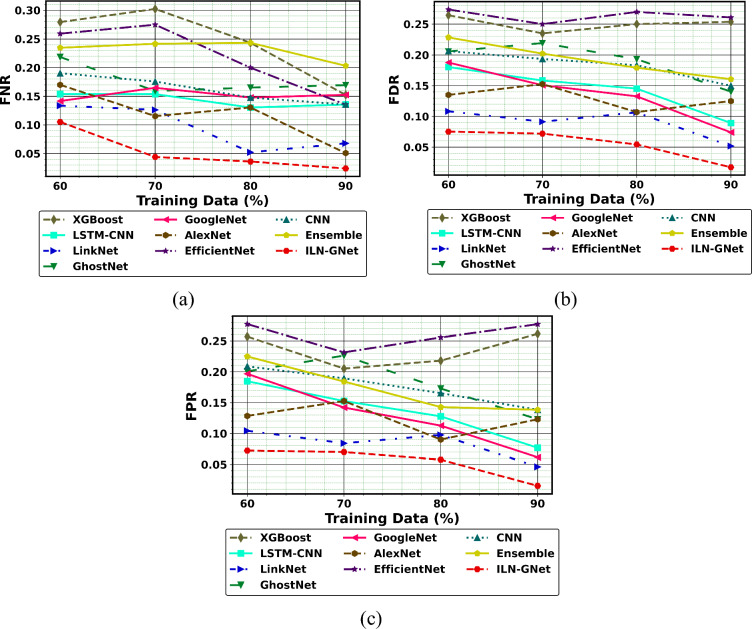
Performance of PD detection with handwriting images using ILN-GNet over traditional method for (**a**) FNR, (**b**) FDR and (**c**) FPR for dataset 2.

Additionally, the performance comparison of the proposed ILN-GNet model over the existing methods such as LinkNet, GhostNet, GoogleNet, AlexNet, EfficientNet, CNN^30^, Ensemble^12^, LSTM-CNN^36^ and XGBoost^46^ using dataset 2 is shown in Fig. 11, Fig. 12 and Fig. 13, respectively. The proposed ILN-GNet model achieved a greater specificity of 0.974 at 80% of training data, which surpasses the results of the traditional methods such as the LinkNet (0.0.950), GhostNet (0.847), GoogleNet (0.853), AlexNet (0.873), EfficientNet (0.804), CNN^30^ (0.849), Ensemble^12^ (0.762), LSTM-CNN^36^ (0.861) and XGBoost^46^ (0.752). Moreover, the suggested ILN-GNet model scored minimal ratings of FNR and FPR across different splits of training data, demonstrating a lower probability of misclassification. Additionally, the suggested ILN-GNet model acquired greater values on F-measure and MCC about 0.961 and 0.974 respectively, in contrast, the existing methods offer lower values on these measures. Therefore, improvements over the conventional methods are largely due to the preprocessing method based on the modified WF, feature extraction based on the modified PHOG, and the integration of the improved LinkNet and GhostNet architecture.

### Ablation analysis

The ablation study employing ILN-GNet for PD identification for datasets 1 and 2, respectively, is displayed in Tables 2 and 3. The performance of ILN-GNet is examined over ILN-GNet with conventional LinkNet, ILN-GNet with conventional LinkNet and Ghostnet, ILN-GNet with conventional PHOG, ILN-GNet with conventional WF, ILN-GNet without feature extraction, Model without MDSCM and Model without WAP-BN. In Table 2, ILN-GNet shows a high accuracy of 0.964, while ILN-GNet with standard LinkNet is 0.931, ILN-GNet with conventional LinkNet and Ghostnet is 0.952, ILN-GNet with standard PHOG is 0.903, ILN-GNet with conventional WF is 0.863, ILN-GNet without extraction of feature is 0.923, Model without MDSCM is 0.893 and Model without WAP-BN is 0.908, displays less accuracy values on dataset 1. In contrast to conventional WF, the modified WF preserves the edges while effectively removing the noise. The enhanced PHOG characteristics aid in gathering the precise details needed for PD identification by increasing the accuracy of gradient magnitude estimation and lessening the effect of higher-frequency noise. Additionally, the changes made to the enhanced LinkNet reduced computation costs and captured aggregate multi-scale context information as compared to the original LinkNet. Particularly, the FDR using ILN-GNet is less than 0.035, while ILN-GNet with conventional LinkNet, ILN-GNet with conventional LinkNet and Ghostnet, ILN-GNet with conventional PHOG, ILN-GNet with conventional WF, ILN-GNet without feature extraction, Model without MDSCM and Model without WAP-BN scores less FDR values.

**Table 2:** Ablation study on ILN-GNet for PD Detection using Dataset 1.

Metrics	ILN-GNet	Model with conventional LinkNet	Model with conventional LinkNet and Ghostnet	Model with conventional PHOG	Model with conventional WF	Model without Feature Extraction	Model without MDSCM	Model without WAP-BN
Accuracy	96.40%	93.10%	95.20%	90.30%	86.30%	92.30%	89.30%	90.80%
Sensitivity	95.70%	92.20%	95.70%	89.60%	84.30%	93.90%	89.10%	91.50%
Specificity	97.00%	94.00%	94.70%	91.00%	88.00%	91.00%	89.50%	90.20%
Precision	96.50%	93.00%	94.00%	89.60%	85.80%	90.00%	87.90%	89.00%
F-measure	96.10%	92.60%	94.80%	89.60%	85.10%	91.90%	88.50%	90.20%
MCC	92.70%	86.20%	90.30%	80.50%	72.40%	84.70%	78.60%	81.60%
NPV	96.30%	93.30%	96.20%	91.00%	86.70%	94.50%	90.60%	92.60%
FPR	3.00%	6.00%	5.30%	9.00%	12.00%	9.00%	10.50%	9.80%
FNR	4.30%	7.80%	4.30%	10.40%	15.70%	6.10%	10.90%	8.50%
FDR	3.50%	7.00%	6.00%	10.40%	14.20%	10.00%	12.10%	11.00%

**Table 3:** Ablation study on ILN-GNet for PD Detection using Dataset 2.

Metrics	ILN-GNet	Model with conventional LinkNet	Model with conventional LinkNet and Ghostnet	Model with conventional PHOG	Model with conventional WF	Model without Feature Extraction	Model without MDSCM	Model without WAP-BN
Accuracy	95.20%	92.30%	94.40%	89.10%	85.10%	91.10%	88.10%	89.60%
Sensitivity	97.40%	94.80%	94.80%	89.60%	87.80%	90.40%	89.10%	89.80%
Specificity	93.20%	90.20%	94.00%	88.70%	82.70%	91.70%	87.20%	89.50%
Precision	92.60%	89.30%	93.20%	87.30%	81.50%	90.40%	85.90%	88.20%
F-measure	94.90%	92.00%	94.00%	88.40%	84.50%	90.40%	87.50%	89.00%
MCC	90.40%	84.80%	88.70%	78.20%	70.40%	82.20%	76.30%	79.20%
NPV	97.60%	95.20%	95.40%	90.80%	88.70%	91.70%	90.20%	91.00%
FPR	6.80%	9.80%	6.00%	11.30%	17.30%	8.30%	12.80%	10.50%
FNR	2.60%	5.20%	5.20%	10.40%	12.20%	9.60%	10.90%	10.20%
FDR	7.40%	10.70%	6.80%	12.70%	18.60%	9.60%	14.10%	11.80%

On observing results in Table 3, the suggested ILN-GNet model’s sensitivity for Dataset 2 is 0.974, which is higher than that of the models with Standard LinkNet (0.948), with Standard LinkNet and GhostNet (0.948), with Standard PHOG (0.896), with Standard WF (0.878), without feature extraction (0.904), with MDSCM (0.891), and with WAP-BN (0.898). With an accuracy of 0.952, the ILN-GNet model has a strong overall detection capability. Notably, the absence of Modified PHOG and Modified WF results in significant accuracy drops to 0.891 and 0.851, respectively. Similarly, the removal of MDSCM and WAP-BN modules causes consistent declines across all performance metrics, emphasizing their importance in enhancing feature representation and classification robustness. These findings underscore the critical role of the Modified Wiener Filter, Modified PHOG-based features, and the enhanced LinkNet architecture incorporating MDSCM and WAP-BN layers in achieving improved detection performance for Parkinson’s Disease.

### Statistical analysis

Tables 4 and 5 detail the statistical analysis in terms of accuracy utilizing ILN-GNet-based PD detection for datasets 1 and 2 respectively. The study displays the improvement of ILN-GNet over LinkNet, GhostNet, GoogleNet, AlexNet, EfficientNet, CNN^30^, Ensemble^12^, LSTM-CNN^36^ and XGBoost^46^. For detecting PD, this work employs improved LinkNet and GhostNet classifiers. Utilizing global information at different scales is greatly aided by the enhanced LinkNet architecture that has been suggested with several structural changes. Furthermore, the improved LinkNet accelerates training and greatly improves operational efficiency. In Table 4, the ILN-GNet reached a higher accuracy of 0.984 for the maximum statistical metric, whereas, LinkNet, GhostNet, GoogleNet, AlexNet, EfficientNet, CNN^30^, Ensemble^12^, LSTM-CNN^36^ and XGBoost^46^ reach lesser accuracies of 0.952, 0.863, 0.903, 0.927, 0.806, 0.871, 0.847, 0.944 and 0.855 respectively. Similarly, a high accuracy of 0.950 is gained for the mean case by ILN-GNet, while LinkNet, GhostNet, GoogleNet, AlexNet, EfficientNet, CNN^30^ and Ensemble^12^ attain lower accuracies. The suggested ILN-GNet model outperformed the existing methods, such as XGBoost (0.760), LSTM-CNN (0.861), LinkNet (0.911), GhostNet (0.821), GoogleNet (0.861), AlexNet (0.880), EfficientNet (0.760), CNN (0.831), and Ensemble model (0.800), as shown by the results in Table 5 for dataset 2. Additionally, when compared to traditional methods, ILN-GNet demonstrated improved classification accuracy with a maximum efficiency value of 0.976. These results demonstrate that the enhancements introduced namely the modified Wiener Filter for preprocessing, modified PHOG-based feature extraction, and the hybrid integration of improved LinkNet with GhostNet collectively contribute to the significant improvement in detection accuracy over traditional methods.

**Table 4:** Statistical study of PD detection with handwriting images using ILN-GNet over traditional methods for dataset 1.

Methods	Mean	Minimum	Median	SD	Maximum
XGBoost	0.824	0.796	0.823	0.024	0.855
LSTM-CNN	0.912	0.883	0.911	0.024	0.944
LinkNet	0.919	0.891	0.916	0.024	0.952
GhostNet	0.829	0.800	0.827	0.025	0.863
GoogleNet	0.870	0.841	0.868	0.025	0.903
AlexNet	0.891	0.861	0.887	0.026	0.927
EfficientNet	0.770	0.742	0.766	0.026	0.806
CNN	0.839	0.810	0.837	0.024	0.871
Ensemble	0.810	0.780	0.806	0.026	0.847
PROP	0.950	0.921	0.947	0.025	0.984

**Table 5:** Statistical study of PD detection with handwriting images using ILN-GNet over traditional methods for dataset 2.

Methods	Mean	Minimum	Median	Std-Dev	Maximum
XGBoost	0.760	0.732	0.759	0.022	0.790
LSTM-CNN	0.861	0.831	0.859	0.024	0.895
LinkNet	0.911	0.881	0.909	0.024	0.944
GhostNet	0.821	0.790	0.819	0.024	0.855
GoogleNet	0.861	0.831	0.859	0.024	0.895
AlexNet	0.880	0.851	0.878	0.023	0.911
EfficientNet	0.760	0.732	0.759	0.022	0.790
CNN	0.831	0.800	0.830	0.024	0.863
Ensemble	0.800	0.770	0.799	0.023	0.831
ILN-GNet	0.942	0.911	0.940	0.024	0.976

### ROC analysis

**Fig.14:**
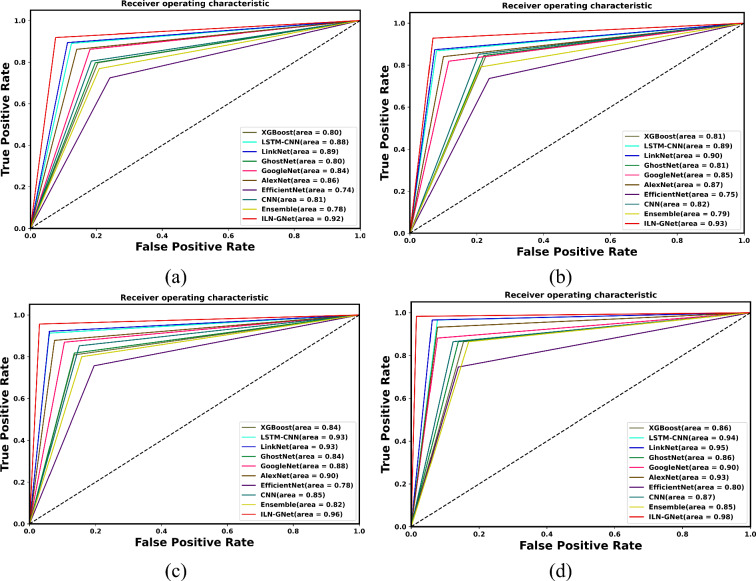
ROC analysis for TD (**a**) 60%, (**b**) 70%, (**c**) 80% and (**d**) 90% for dataset 1.

**Fig.15:**
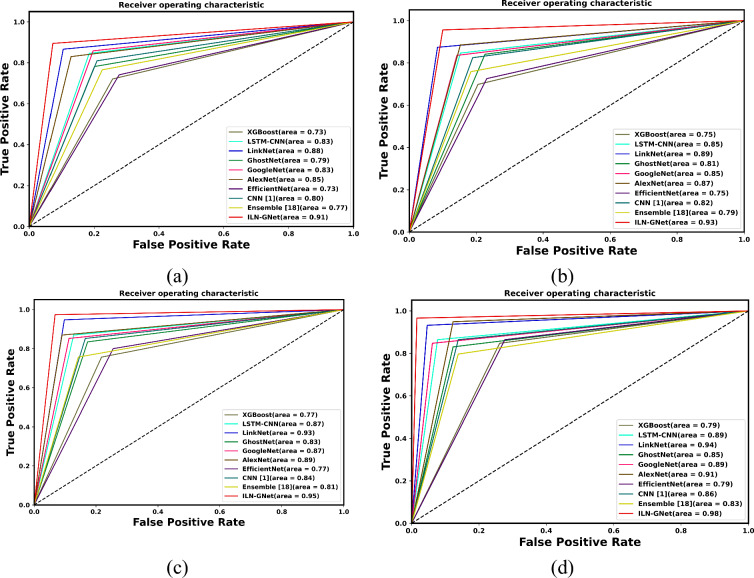
ROC analysis for TD (**a**) 60%, (**b**) 70%, (**c**) 80% and (**d**) 90% for dataset 2.

Figure 14 and Fig. 15 express the ROC analysis for varied TD, such as 60, 70, 80 and 90 using datasets 1 and 2 respectively. It is observed that for all TDs, the proposed ILN-GNet attains high TPR values when compared to others. Particularly, the proposed ILN-GNet attains a high TPR of 1.0 for all TDs when FPR is 1.0 for dataset 1. The AUC values for the proposed ILN-GNet are 0.92, 0.93, 0.96 and 0.98 for TDs 60, 70, 80 and 90, respectively. Thus, the ROC plot shows the performance of the proposed ILN-GNet over LinkNet, GhostNet, GoogleNet, AlexNet, EfficientNet, CNN^30^, Ensemble^12^, LSTM-CNN^36^ and XGBoost^46^ models with high TPR values. For dataset 2, at 70% of training data, the ILN-GNet model scored better values on TPR about 0.96 and FNR about 0.1. The AUC value of the ILN-GNet method is 0.98. the findings show that the suggested ILN-GNet model scored higher TPR demonstrating fewer false negatives and lower FPR scores, suggesting that the model contributes to minimal misclassified cases. These results demonstrate the suggested ILN-GNet model precisely classified the healthy and patients in contrast to the existing methods.

### Confusion matrix

**Fig.16:**
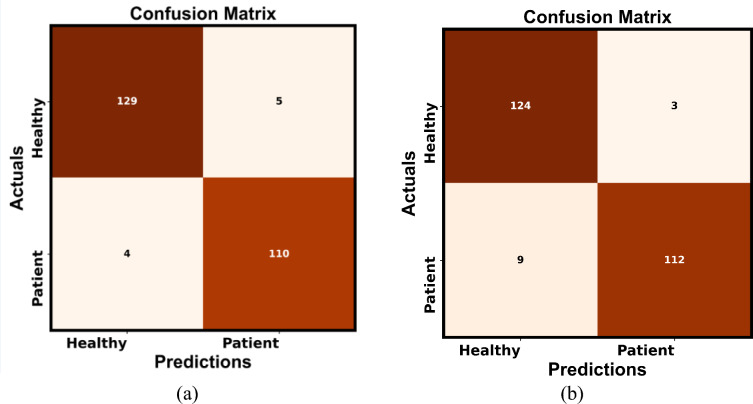
Confusion matrix using proposed ILN-GNet for PD detection for (**a**) Dataset 1 and (**b**) Dataset 2.

The confusion matrix given in Fig. 16 aids in evaluating the classification efficiency of the DL model for datasets 1 and 2. It distinguishes the predicted output by DL from the actual targeted values. The confusion matrix contains the values of TP, FP, FN and TN. Using the proposed ILN-GNet for PD detection at 80% TD exhibits TP = 129, FP = 5, FN = 4 and TN = 110, respectively. This indicates that five healthy individuals’ handwriting was incorrectly classified as PD-affected. This suggests that the model may be overly sensitive to certain handwriting characteristics that are shared between healthy and PD samples. Using dataset 2, the ILN-GNet model achieved TP = 124 and FN = 3, which indicates that the PD-affected individuals were incorrectly classified as healthy. Overall, the ILN-GNet model efficiently detects the healthy and patient classes.

### Analysis of computational time

Computational time analysis is the process of determining how long a model will take to complete a task. It is a crucial performance indicator that assesses the efficacy of the model. The proposed ILN-GNet model’s computational time comparison with existing methods, such as EfficientNet, Ensemble, CNN, LSTM-CNN, LinkNet, XGBoost, GhostNet, GoogleNet, and AlexNet utilizing datasets 1 and 2, is shown in Table 6. As a result, the suggested ILN-GNet method demonstrates superior efficiency in terms of processing speed. ILN-GNet recorded minimal computational times of 63.748 s on Dataset 1 and 62.355 s on Dataset 2, outperforming all other compared models. In contrast, the LSTM-CNN required significantly more time, with 128.650 s for dataset 1 and 125.650 s for dataset 2, and LinkNet took 107.111 s on dataset 1 and 106.452 s on dataset 2. Even relatively lightweight architectures such as GhostNet, GoogleNet, and EfficientNet demonstrated higher computation times compared to ILN-GNet, with GhostNet being the closest in efficiency at 74.134 s and 73.658 s, still slower. Traditional models like XGBoost also lag behind, requiring over 100 s on both datasets. These results confirm that ILN-GNet not only delivers high precision, as previously shown but also maintains a low computational burden, making it highly suitable for real-time PD detection applications.

**Table 6:** Analysis of computational time for datasets 1 and 2.

Methods	Dataset 1	Dataset 2
	Computational Time (s)	Computational Time (s)
XGBoost	105.680	103.587
LSTM-CNN	128.650	125.650
LinkNet	107.111	106.452
GhostNet	74.134	73.658
GoogleNet	88.958	85.649
AlexNet	85.521	83.652
EfficientNet	84.165	86.568
CNN	96.555	97.688
Ensemble	101.466	102.697
ILN-GNet	63.748	62.355

### Analysis of cross-validation

An important machine learning technique is cross-validation, which tests a model on several dataset subsets to assess its generalizability and resilience. This study evaluates the effectiveness of the proposed ILN-GNet model using two datasets, Dataset 1 and Dataset 2. The cross-validation results of the ILN-GNet model for these datasets are presented in Table 7 and Table 8, respectively. The model’s efficiency is validated using standard measures such as specificity, accuracy, sensitivity, precision, MCC, NPV, F-measure, FNR, and FPR. For Dataset 1 (trained on Dataset 1 and tested on Dataset 2), the ILN-GNet model achieved an accuracy of 0.951 and a precision of 0.914, demonstrating its high reliability in distinguishing between healthy and affected individuals. Similarly, the model performed even better in several areas for Dataset 2 (trained on Dataset 2 and tested on Dataset 1), obtaining a sensitivity of 0.970 and an NPV of 0.969, demonstrating a significant capacity to accurately detect true positive and true negative cases. Furthermore, the model maintained low error rates, with an FNR of 0.030 and FPR of 0.030, signifying a minimal rate of missed or incorrect predictions. These cross-validation results show that the proposed ILN-GNet model performs better in PD identification and has good generalizability and reliability across two datasets.

**Table 7:** Analysis of Cross-validation for Dataset 1.

Measures	Training with Dataset 1 and Testing with Dataset 2	Training with Dataset 2 and Testing with Dataset1
Accuracy	95.10%	95.20%
Sensitivity	96.40%	96.50%
Specificity	92.10%	92.20%
Precision	91.40%	91.50%
F-measure	93.70%	93.80%
MCC	90.20%	90.40%
NPV	96.20%	96.40%
FPR	7.90%	7.80%
FNR	3.60%	3.50%
FDR	8.60%	8.50%

**Table 8:** Analysis of Cross-validation for Dataset 2.

Measures	Training with Dataset 1 and Testing with Dataset 2	Training with Dataset 2 and Testing with Dataset1
Accuracy	94.80%	94.90%
Sensitivity	96.90%	97.00%
Specificity	92.50%	92.60%
Precision	91.30%	91.30%
F-measure	93.30%	93.40%
MCC	89.20%	89.30%
NPV	96.80%	96.90%
FPR	7.50%	7.40%
FNR	3.10%	3.00%
FDR	8.70%	8.70%

### Analysis of statistical test

The results of the statistical test establish if the suggested ILN-GNet model’s improvements over traditional techniques are statistically significant. The T-test, Friedman, and Wilcoxon tests were used in this study to evaluate the ILN-GNet model’s performance against the results of traditional techniques.

The Wilcoxon test results, which compare the efficiency of the suggested ILN-GNet model to other approaches across two datasets, are shown in Table 9. A p-value less than 0.1 indicates statistical significance, suggesting a significant performance difference between ILN-GNet and the respective models. For Dataset 1, ILN-GNet demonstrates statistically significant improvements over XGBoost, LSTM-CNN, LinkNet, GhostNet, GoogleNet, AlexNet, EfficientNet, and Ensemble, with p-values consistently below 0.1, except for CNN (p-value = 0.100), which shows no significant difference. On Dataset 2, ILN-GNet outperforms most models, including XGBoost, LSTM-CNN, GoogleNet, AlexNet, and EfficientNet, with p-values ranging from 0.004 to 0.082, indicating clear statistical significance. The Wilcoxon test highlights ILN-GNet’s superior performance across both datasets, with the model’s architecture leading to more accurate and robust PD detection, particularly in comparison to models like CNN and LinkNet, which show no significant advantage over ILN-GNet. Overall, the results suggest that the hybrid design of ILN-GNet is highly effective for PD detection tasks.

**Table 9:** Analysis of Wilcoxon test using datasets 1 and 2.

ILN-GNet model Vs	Dataset 1	Dataset 2
	Wilcoxon p-value	Wilcoxon p-value
XGBoost	0.065	0.008
LSTM-CNN	0.032	0.062
LinkNet	0.048	0.100
GhostNet	0.082	0.082
GoogleNet	0.056	0.032
AlexNet	0.076	0.011
EfficientNet	0.022	0.004
CNN	0.100	0.065
Ensemble	0.051	0.067

The Friedman test results are shown in Table 10, which contrasts the suggested ILN-GNet model’s performance with that of several alternative models for Datasets 1 and 2. A p-value less than 0.1 indicates statistical significance, suggesting a significant performance difference between ILN-GNet and the respective models. For Dataset 1, ILN-GNet shows statistically significant improvements over LSTM-CNN (p-value = 0.037), EfficientNet (p-value = 0.022), XGBoost (p-value = 0.082), LinkNet (p-value = 0.061), and GoogleNet (p-value = 0.072) approach significance. For Dataset 2, ILN-GNet outperforms XGBoost (p-value = 0.005), EfficientNet (p-value = 0.002), and AlexNet (p-value = 0.008), showing stronger statistical significance. Other models like LSTM-CNN (p-value = 0.078), GoogleNet (p-value = 0.037), and Ensemble (p-value = 0.083) show some level of significance. Models such as CNN, and LinkNet do not show significant performance differences from ILN-GNet on either dataset. Overall, the Friedman test confirms that ILN-GNet consistently demonstrates superior performance, particularly on Dataset 2, underscoring its effectiveness in detecting Parkinson’s Disease across different model comparisons.

**Table 10:** Analysis of Friedman test for Dataset 1 and Dataset 2.

ILN-GNet model Vs	Dataset 1	Dataset 2
	Friedman p-value	Friedman p-value
XGBoost	0.082	0.005
LSTM-CNN	0.037	0.078
LinkNet	0.061	0.100
GhostNet	0.095	0.095
GoogleNet	0.072	0.037
AlexNet	0.091	0.008
EfficientNet	0.022	0.002
CNN	0.100	0.082
Ensemble	0.065	0.083

The T-test results comparing the efficiency of the suggested ILN-GNet model with several different models across Datasets 1 and 2 are shown in Table 11. A p-value less than 0.1 indicates statistical significance, suggesting a significant performance difference between ILN-GNet and the respective models. For Dataset 1, ILN-GNet shows statistically significant superiority over EfficientNet (p-value = 0.045) and XGBoost (p-value = 0.080), as both p-values are below 0.1, indicating a meaningful performance gap. Models like LSTM-CNN (p-value = 0.070), GoogleNet (p-value = 0.080), and Ensemble (p-value = 0.070) also show a significant difference, although the performance gap is smaller. Other models such as LinkNet (p-value = 0.080), GhostNet (p-value = 0.090), AlexNet (p-value = 0.090), and CNN (p-value = 0.100) show no significant performance difference, with p-values approaching 0.1. For Dataset 2, ILN-GNet shows statistically significant improvement over EfficientNet (p-value = 0.016), XGBoost (p-value = 0.025), and AlexNet (p-value = 0.045), with all these models having p-values below 0.1, indicating that ILN-GNet performs better. GoogleNet (p-value = 0.061) also shows a significant difference, though not as strong as the previous models. In contrast, models like LSTM-CNN (p-value = 0.080), Ensemble (p-value = 0.080), and CNN (p-value = 0.080) have p-values near 0.1, which indicate marginal but not conclusive differences in performance. Overall, the T-test results show that ILN-GNet significantly outperforms several models on both datasets.

**Table 11:** Analysis of T-test for datasets 1 and 2.

ILN-GNet model Vs	Dataset 1	Dataset 2
	T-test	T-test
XGBoost	0.080	0.025
LSTM-CNN	0.070	0.08
LinkNet	0.080	0.1
GhostNet	0.090	0.09
GoogleNet	0.080	0.061
AlexNet	0.090	0.045
EfficientNet	0.045	0.016
CNN	0.100	0.08
Ensemble	0.070	0.08

## Conclusion

This research developed a novel PD detection approach with handwriting images using an improved hybrid classification model. The handwriting image was pre-processed primarily using modified WF. Next, deep features, shape features, and modified PHOG were obtained. Lastly, a hybrid ILN-GNet framework was used for detection, and its average indicated whether the person was affected or healthy. Additionally, the effectiveness of the proposed ILN-GNet model is contrasted with conventional techniques like EfficientNet, LinkNet, GhostNet, GoogleNet, and AlexNet as well as cutting-edge models like CNN and Ensemble. Thus, at 90% of training data, the proposed ILN-GNet model had a higher accuracy of 0.982, while LinkNet, GhostNet, GoogleNet, AlexNet, EfficientNet, CNN, and Ensemble had an accuracy of 0.921, 0.864, 0.916, and 0.827, respectively. The F-measure and MCC of the suggested ILN-GNet model were higher, at 0.966 and 0.971, respectively. The incorporation of the modified wiener filter, the extraction of improved PHOG along the retrieval of deep features and shape features contribute to enhanced detection. Additionally, the Improved LinkNet architecture is proposed with structural modifications using an MDSCM layer and integrated with GhostNet. The resulting hybrid model demonstrates significantly better performance compared to existing methods.

Despite the promising results, this study has several limitations. The performance of the modified Wiener filter used in pre-processing is critical; improper tuning may lead to the loss of important handwriting features. Furthermore, the combination of Improved LinkNet with GhostNet raises the model’s overall complexity even though it might improve classification accuracy. In order to improve the model’s performance even more, future studies will concentrate on resolving these issues. Future research would examine improving preprocessing methods to lower false positives. Only the most pertinent features are chosen, which helps keep the framework from overfitting to unimportant data and increases the model’s overall accuracy by concentrating on the most significant patterns. For this purpose, optimal feature selection techniques could be explored in future studies. Moreover, future studies will explore alternative ML classifiers and fusion strategies such as weighted fusion to assess and improve the overall performance of the model. Furthermore, recognizing that PD has no cure but that early intervention can significantly alleviate symptoms, our future work’s aim is to integrate the proposed approach into the healthcare environment with the support of medical professionals and to enhance the model to assess symptom severity and eventually provide personalized treatment suggestions such as medication options, physical therapy, or speech therapy, based on patient data.

## Data Availability

The data underlying this article are available in the Kaggle repository named as HandPD dataset, at https://www.kaggle.com/datasets/claytonteybauru/spiral-handpd/data and theMeander_HandPD images in HandPD dataset at https://wwwp.fc.unesp.br/ ~ papa/pub/datasets/Handpd/dataset, at https://www.kaggle.com/datasets/claytonteybauru/spiral-handpd/data and theMeander_HandPD images in HandPD dataset athttps://wwwp.fc.unesp.br/~papa/pub/datasets/Handpd/

## Abbreviations

A-LSTM: Attention-based long short-term memory
BN: Batch normalization
Conv: Convolution
CC-Net: Continuous convolution network
CNN: Convolutional neural networks
DT: Decision trees
DL: Deep learning
DBS: Deep brain stimulation
DCNN: Deep convolutional neural network
DNN: Deep neural networks
DSS: Decision support systems
ELU: Exponential linear unit
FCL: Fully connected layer
FGS: Fuzzy genetic systems
FC: Fuzzy classifier
GNet: Ghostnet
GA: Genetic algorithms
GBM: Ghost bottleneck module
GSA: Grid search algorithm
ILN-GNet: Improved linknet and ghostnET
KNN: K-nearest neighbor
LR: Logistic regression
LS-SVR v: Least squares support vector regression
MDSCM: Multisize depth wise separable convolution module
ML: Machine learning
NB: Naïve bayes
PSD: Power spectral density
PHOG: Pyramid histogram of oriented gradients
PCA: Principal component analysis
PD: Parkinson’s disease
QMFOFS-HCNN: Quantum mayfly optimization-based feature subset selection with hybrid convolutional neural network
QMO: Quantum mayfly optimization
RF: Random forest
SD: Standard deviation
SVC: Support vector classification
TL: Transfer learning
TD: Training data
UPDRS: Unified parkinson’s disease rating scale
WF: Wiener filter
WAP-BN: Weighted average pooling-batch normalization
